# Treatment of Wounds of the Pelvic Basin

**Published:** 1918-08

**Authors:** 


**Treatment of Wounds of the Pelvic Basin: Especially of the Bladder and Rectum.** By M. J. Tanton. Report before the Second Session of Interallied Surgical Conference.

In this very important article Tanton discusses :

A. *Isolated Wounds of the Bony Pelvis.* These represent about 1.06 o/o of all war wounds. In examination of recent wounded (3716 new cases as well as 414 old ones) the lesions were distributed as follows :

| | Recent : | Old : |
|---|---|---|
| Iliac Bodies | 1659 | 199 |
| Iliac Crests | 311 | 43 |
| Sacrum | 652 | 62 |
| Coccyx | 24 | 1 |
| Sacrum and Coccyx | 21 | 4 |
| Wing of Sacrum | 20 | 2 |
| Sacro Iliac area | 120 | 41 |
| Pubic Bone | 202 | 12 |
| Pubic symphysis | 12 | 2 |
| Pubo-ischial ramus | 47 | 10 |
| Ischium | 241 | 29 |
| Ilium and others | 60 | 9 |

(Note : In a large number of lesions the exact site was not mentioned.)

These lesions may be most complicated and serious, and may be associated with injury to the urethra, retroperitoneal suppurations, opening and infection of the meninges, severe hemorrhage, especially following gluteal artery injuries, and injury to the sacral plexus and its branches. Serious infection, especially gas gangrene, is common. Chronic osteomyelitis is the usual sequela.

*Treatment* : Debridement in early cases. Excision with well arranged drainage in late cases.

B. *Wounds of the Bony Pelvis and of the Bladder* : 367 Cases. Of 334 recent cases 68 intraperitoneal and 266 extraperitoneal vesical injuries. With intraperitoneal injuries other viscera are often damaged. In severe cases a traumatic extrophy may result.

Treatment of intraperitoneal cases. Debridement of the whole wound with sutures in layers of the hole in the bladder (and other organs). If bladder cannot be completely closed, the nonsutured part should be extraperitonealized (with suprapubic drainage). In completely closed cases, permanent catheter or regular catheterization may be used. (Recommends drain in Douglas for two to three days).

Extraperitoneal cases are more difficult to treat. There should be surgical excision of wound, primary suprapubic cystostomy. Some cases heal spontaneously with urine leaking through wound or wounds. If serious fractures are present; infection is likely and grave consequences may develop if the urine has not been side-tracked by cystostomy.

External urinary fistulae and osteomyelitic fistulae are frequent sequelae. Internal vesico-bone fistulae also occur. These may lead to calculi. Ascending pyelonephritides are quite common.

C. *Wounds of the Rectum with or without Lesions of Bony Pelvis:* 517 Cases.

273 cases rectum and bony pelvis involved (sacrum and coccyx, less frequently sacroiliac area; ischium; wing of ilium and acetabulum). Extraperitoneal; serious infections, extra or intraperitoneal, are very liable to develop, though early operation (débridement with or without closure of the tears) leaving the whole wound wide open, may lead to excellent results. Some advise artificial iliac anus as routine. Others recommend constipation with opium. Stercoral fistulae are common sequelae but they usually heal. Sphincter disturbances are common.

D. *Associated Wounds of Bladder and of Rectum*: 224 cases.

Intraperitoneal are much less frequent. Laparatomy is indicated. In 16 cases, 14 died.

Extraperitoneal are either vesicorectal or anaurethral (prostatic urethra).

Treatment is directed against passage of feces by rectal wounds and into bladder, as well as prevention of infection. Wound in rectum should be sutured or the anterior rectal wall should be lowered (pulled down) so as to block the vesical opening. The bladder should be drained by catheter (in dwelling) or cystostomy (suprapubic). Colostomy has but few adherents while cystostomy is getting more popular. The author is inclined to avoid the latter and do the former. Subsequent plastic operations may be required for stenoses and prolapse incontinence as well as for chronic fistulae.

